# Estimation of intraoperative blood loss in hepatopancreatobiliary surgery: a Delphi consensus process of the European–African Hepato-Pancreato-Biliary Association (E-AHPBA)

**DOI:** 10.1093/bjs/znae256

**Published:** 2024-10-10

**Authors:** Giampaolo Perri, Ernesto Sparrelid, Ajith K Siriwardena, Giovanni Marchegiani, E-AHPBA Consensus Group on Blood Loss in Hepatopancreatobiliary Surgery, Mohamad Abu Hilal, Mustapha Adam, Luca Aldrighetti, Bodil Andersson, Angelakoudis Apostolos, Somaiah Aroori, Anita Balakrishnan, Domenico Bassi, Andrea Belli, Giammauro Berardi, Frederik Berrevoet, Marc Besselink, Maximilian Bockhorn, Ugo Boggi, René Borscheid, Stefan A W Bouwense, Raffaele Brustia, Carljin Buis, Sean Burmeister, Olivier Busch, Umberto Cillo, Ahmet Coker, Kevin Conlon, Stefano Crippa, Francesco E D'Amico, Bobby V M Dasari, Raffaele De Luca, Roeland F De Wilde, Christos Dervenis, John Devar, Marcello Di Martino, Safi Dokmak, Ela Ekmekcigil, Jennie Engstrand, Alessandro Esposito, Asmund Fretland, Isabella Frigerio, Tom Gallagher, Georgios Gemenetzis, Stefan Gilg, Francesco Giovinazzo, Brian K P Goh, Martina Guerra, Rachel Guest, Aiste Gulla, Andrew A Gumbs, Thilo Hackert, Julie Hallet, Fiona Hand, Daniel Hartmann, Andrew J Healey, Stefan Heinrich, Emir Hoti, Povilas Ignatavicius, Nigel Jamieson, Laukkarinen Johanna, Ed Jonas, Elio Jovine, Ben Jugmohan, Ambareen Kausar, Elie Keli, Zafar Khan, Jakob Kirkegard, Jorg Kleeff, Philipp Kron, Francesco Lancellotti, Sven Lang, Uttam Laudari, Johanna Laukkarinen, Michael Linecker, Victor Lopez, Hassan Z Malik, Alessio Marchetti, Guillaume Martel, Emmanuel Melloul, Chistoph Michalski, Sanjay Pandanaboyana, Ioannis Passas, Julie Perinel, Dejan Radenkovic, Jose M Ramia, Elena Rangelova, Niki Rashidian, Francesca Ratti, Artur Rebelo, Rami Rhaiem, Fernando Rotellar, Eran Sadot, Ville Sallinen, Tsaramanidis Savvas, Moritz Schmelzle, Alejandro Serrablo, Mario M Serradilla, Olivia Sgarbura, Erik Shadde, Kjetil Soreide, Carlo Sposito, Stefan Stattner, Gregor A Stavrou, Hanna Sternby, Oliver Strobel, Christian Sturesson, Lulu Tanno, Michele Tedeschi, Guido Torzilli, Gregory Tsiotos, Georgios Tsoulfas, Patricia S Velazquez

**Affiliations:** Hepato-Pancreato-Biliary and Liver Transplant Surgery Unit, Department of Surgical, Oncological and Gastroenterological Sciences (DiSCOG), University of Padua, Padua, Italy; Department of General Surgery, IRCCS Azienda Ospedaliero-Universitaria di Bologna, Maggiore Hospital, Bologna, Italy; Department of Clinical Science, Intervention and Technology, Division of Surgery, Karolinska University Hospital, Stockholm, Sweden; Hepatobiliary and Pancreatic Surgery Unit, Manchester Royal Infirmary, Manchester University NHS Foundation Trust, Manchester, UK; Hepato-Pancreato-Biliary and Liver Transplant Surgery Unit, Department of Surgical, Oncological and Gastroenterological Sciences (DiSCOG), University of Padua, Padua, Italy

## Introduction

Intraoperative blood loss is considered a key quality indicator and outcome metric in hepatopancreatobiliary (HPB) surgery^1–3^. The literature also suggests that blood loss could be correlated with morbidity and mortality, either directly or as a surrogate marker^4–8^. Blood loss is a common primary endpoint when introducing new or minimally invasive surgical techniques in HPB surgery^9^, and potentially one of the few actionable risk factors for morbidity. There are, however, no specific benchmarks nor standardization of practice regarding its intraoperative assessment. The range of available approaches and the absence of a method identified as superior has led to inconsistency and heterogeneity in the practice of blood loss estimation, in general as well as in HPB surgery^10,11^. According to a recent systematic review^9^, most studies reporting intraoperative blood loss during liver and/or pancreatic surgery did not specify the estimation method used. Among the minority of studies reporting it, more than nine different options were described. Consequently, most of the available evidence proving a significant association between blood loss and perioperative outcomes did not include an empirical and systematic calculation of blood loss. This affects the reproducibility of the results, and prevents an objective grading of the clinical importance of blood loss. The adoption of a universal, standard estimation method is a necessary step to ensure consistent reporting. In the absence of published guidance, the aim of this project was to identify areas of consensus regarding definition and estimation of intraoperative blood loss in HPB surgery among international experts from the European–African Hepato-Pancreato-Biliary Association (E-AHPBA).

## Methods

### Overview

This Delphi consensus project, supported by the scientific research committee of the E-AHPBA, was intended as practical guide to standardize intraoperative blood loss estimation for HPB surgery. The scope of the project was to obtain consensus on a definition of intraoperative blood loss and optimal estimation method. The final recommendations are based on expert consensus; therefore, grading of evidence levels was not used^12^, and this report is not to be considered as a comprehensive evidence review.

### Participants

Consensus participants were selected as follows: surgeons who had published work in areas relevant to the consensus; surgeons with an international reputation for high-level experience in HPB surgery; and surgeons who declared their interest in participating while attending the symposium dedicated to the consensus, held at the 15th biennial conference of the E-AHPBA in Lyon, France, on 17 June 2023. Participants in the consensus comprised 109 clinicians, distributed within the European territory, who are included in the present study as collaborators.

### Statements

The steering committee (G.P., E.S., A.K.S., and G.M.) produced a draft of 13 statements covering the scope of the project. The first draft of the statements was based on a previously published systematic review^9^, including a search in PubMed for original studies, published before October 2021, that reported the estimated intraoperative blood loss of patients undergoing pancreatic or hepatic resections. The results of a previous worldwide snapshot survey^9^ administered to surgeons performing pancreatic and/or liver surgery, and validation of the findings of the systematic review, were also taken into account. The 13 statements comprised 5 different subgroups: importance of intraoperative blood loss estimation^1,2^; definition of estimated blood loss^3^; methods of blood loss estimation^4–8^; blood loss estimation and existing bleeding scales^9,10^; and blood loss grading and clinical relevance^11–13^.

### Consensus process

The consensus process took place between June and December 2023, and consisted of three phases: definition of domains to be addressed in the consensus by the steering committee, after the systematic review and discussion on a face-to-face community consultation; drafting of the statements; and one round of a Delphi process^13^. The face-to-face community consultation was held at the dedicated symposium during the aforementioned E-AHPBA conference. Drafts of the 13 statements provided by the panel of experts were discussed individually and modified according to audience comments during the meeting. Once developed, the final questionnaire was distributed online for the Delphi process to the consensus participants, with an individualized e-mail invitation including a link to a secure website. The first round took place between 16 October and 13 November 2023 (with 2 e-mail reminders before closure of the voting round). The consensus participants were asked to rate their agreement or disagreement with each statement using a five-point Likert scale (1, strongly disagree; 2, disagree; 3, agree; 4, more than agree; 5, strongly agree). The sum of points 1–2 was considered ‘negative consensus’, whereas the sum of points 3–5 was considered ‘positive consensus’. Anonymous responses to the items were tabulated into a centralized database and analysed by the expert panel after closure of the round. A cut-off of 80% agreement/disagreement for each statement was deemed necessary to define consensus. No consensus was reached if a statement received less than 80% concordant replies. The results of the first round of voting formed the basis for the current consensus. The results of the Delphi round were shared online with all consensus participants for comments between 27 November and 31 December 2023. Persisting controversial aspects were noted and acknowledged in the final document. The online platform for the Delphi round was provided by a professional healthcare consulting company (https://ethossrl.com).

### Ethics

No institutional ethics committee review was required as this study involved no patient contact. Implied consent was assumed based on the voluntary responses of the participants.

## Results

**Fig. 1:**
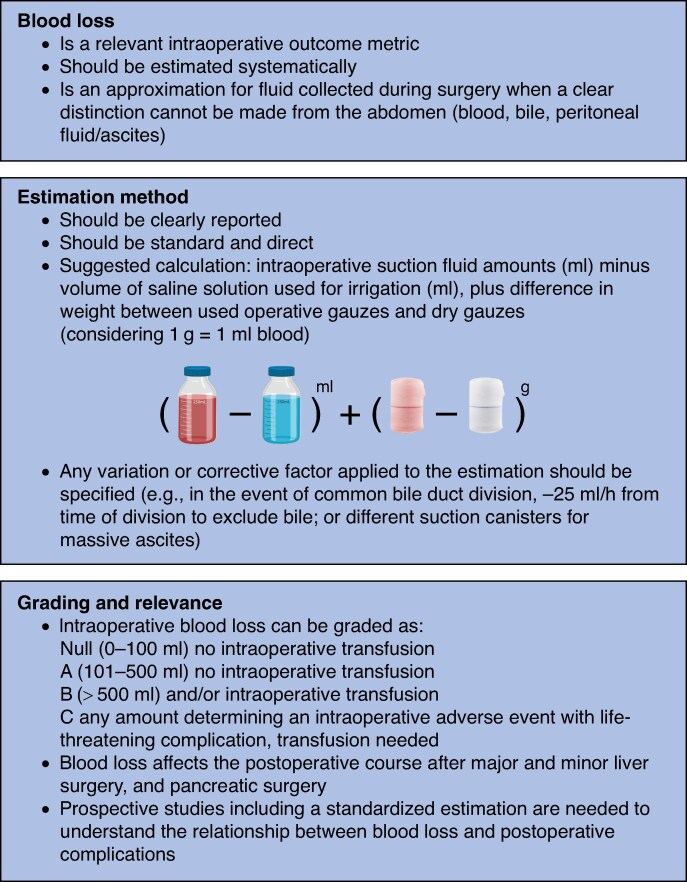
Summary of E-AHPBA consensus statements for intraoperative blood loss in hepatopancreatobiliary surgery

Of 122 HPB surgeons invited, 109 (89.3%) participated in the Delphi round. All statements reached positive consensus (>80%) after the first round, with consensus of at least 90% for 11 of 13 statements. The minimum level of consent was 80.8% (statement 3). The 13 final statements with respective agreement levels are shown in *Table 1* and summarized in *Fig. 1*.

**Table 1:** E-AHPBA consensus statements for intraoperative blood loss in hepatopancreatobiliary surgery

	No. of participants					
	Score					Total
	1	2	3	4	5	
**Importance of intraoperative blood loss**						
** **Statement 1—Blood loss is a relevant intraoperative outcome metric in HPB surgery	0	2	11	18	78	109
	(1.8%)		(98.2%)			
** **Statement 2—Blood loss should be estimated systematically	0	1	10	21	77	109
	(0.9%)		(99.1%)			
**Definition of estimated blood loss**						
** **Statement 3—Estimated blood loss represents an approximation not necessarily referring to blood only, but to the fluid collected during surgery from the abdomen (blood, bile, peritoneal fluid/ascites) when a clear distinction cannot be made	4	17	20	26	42	109
	(19.2%)		(80.8%)			
**Methods of blood loss estimation**						
** **Statement 4—Method of blood loss estimation should be clearly reported	0	2	11	31	65	109
	(1.8%)		(98.2%)			
** **Statement 5—A standard, direct method for estimation of blood loss should be applied to ensure consistent reporting and reproducibility	0	0	8	27	74	109
	(–)		(100%)			
** **Statement 6—Suggested direct estimation method for blood loss is a calculation based on intraoperative suction fluid amounts (ml) minus volume of saline solution used for irrigation (ml), plus difference in weight between used operative gauzes and dry gauzes (considering 1 g = 1 ml blood)	0	5	31	41	32	109
	(4.5%)		(95.5%)			
** **Statement 7—Any variation or corrective factor applied to the estimation should be specified (for example, in the event of common bile duct division, −25 ml/h from the time of division to exclude bile; or different suction canisters for massive ascites)	1	5	40	28	35	109
	(5.5%)		(94.5%)			
** **Statement 8—Indirect estimation methods (for example, blood count-related formulas) are encouraged to complement direct estimation and for research purposes, but do not replace direct estimation	0	7	37	36	29	109
	(6.4%)		(93.6%)			
**Blood loss estimation and existing bleeding scales**						
** **Statement 9—Use of existing tools to measure intraoperative bleeding from a specific site (for example VIBe scale) complements, but does not replace, systematic estimation of whole blood loss	1	1	44	30	33	109
	(1.8%)		(98.2%)			
** **Statement 10—Clinical trials evaluating grade of bleeding treated, assessing haemostasis, should adopt an intraoperative bleeding scale (for example, VIBe scale)	0	5	44	31	29	109
	(4.5%)		(95.5%)			
**Blood loss grading and clinical relevance**						
** **Statement 11— Intraoperative blood loss can be graded, based on quantity and severity: null (0–100 ml), no intraoperative transfusion; A (101–500 ml), no intraoperative transfusion; B (> 500 ml), and/or intraoperative transfusion; C, any amount determining an intraoperative adverse event with life-threatening complication, transfusion needed	5	10	34	32	28	109
	(13.7%)		(86.3%)			
** **Statement 12—Intraoperative blood loss affects the postoperative course after:						
12.1 Pancreatic surgery	2	2	20	24	61	109
	(3.6%)		(96.4%)			
12.2 Major liver surgery	2	0	14	20	73	109
	(1.8%)		(98.2%)			
12.3 Minor liver surgery	2	7	40	35	25	109
	(8.2%)		(91.8%)			
** **Statement 13—Further prospective studies including a standardized estimation are needed to understand the relationship between blood loss and postoperative complications after HPB surgery	0	1	9	34	65	109
	(0.9%)		(99.1%)			

Values in parentheses are % negative (scores 1–2) and positive (score 3–5) consensus. HPB, hepatopancreatobiliary; VIBe scale, validated intraoperative bleeding severity scale.

### Importance of intraoperative blood loss

According to the panel, blood loss is a relevant intraoperative outcome metric in HPB surgery (statement 1: 98.2% agreement) and should be estimated systematically (statement 2: 99.1% agreement).

### Definition of estimated blood loss

The panel defined estimated blood loss as an approximation, referring not necessarily to blood only, but to fluid collected from the abdomen during surgery (blood, bile, peritoneal fluid/ascites) when a clear distinction cannot be made (statement 3: 80.8% agreement).

### Methods of blood loss estimation

The panel agreed that the method of blood loss estimation during surgery should be clearly reported (statement 4: 98.2% agreement). A standard, direct method for estimation should be applied to ensure consistent reporting and reproducibility (statement 5: 100% agreement). The direct estimation method suggested by the panel is calculation based on intraoperative suction fluid amounts (ml) minus volume of saline solution used for irrigation (ml), plus the difference in weight between used operative gauzes and dry gauzes (considering 1 g is equivalent to 1 ml blood) (statement 6: 95.5% agreement). Any variation or corrective factor applied to the estimation should be specified (for example, in the event of common bile duct division, −25 ml/h from the time of division to exclude bile) (statement 7: 94.5% agreement). The panel encourages the use of indirect estimation methods (for example, blood count-related formulas) to complement direct estimation and for research purposes, but not as replacement for direct estimation (statement 8: 93.6% agreement).

### Blood loss estimation and existing bleeding scales

According to the panel, the use of existing tools to measure intraoperative bleeding from a specific site (for example, validated intraoperative bleeding severity (VIBe) scale^14^) complements, but does not replace, systematic estimation of whole blood loss (statement 9: 98.2% agreement). Clinical trials evaluating the grade of bleeding treated, assessing haemostasis, should adopt an intraoperative bleeding scale (for example, VIBe scale^14^) (statement 10: 95.5% agreement).

### blood loss grading and clinical relevance

The panel recommends intraoperative blood loss to be graded as follows, based on quantity and severity: null (0–100 ml), no intraoperative transfusion; A (101–500 ml), no intraoperative transfusion; B (over 500 ml), and/or intraoperative transfusion; and C, any amount determining an intraoperative adverse event with life-threatening complication, transfusion needed (statement 11: 86.3% agreement) (*Table 2*). The panel acknowledges that intraoperative blood loss affects the postoperative course after pancreatic surgery (statement 12.1: 96.4% agreement), and both major (statement 12.2: 98.2% agreement) and minor (statement 12.3: 91.8% agreement) liver surgery. However, further prospective studies including a standardized estimation are needed to understand the relationship between blood loss and postoperative complications after HPB surgery (statement 13: 99.1% agreement).

**Table 2:** E-AHPBA consensus definition and grading of estimated blood loss in hepatopancreatobiliary surgery

Definition of blood loss	Estimation method	Grade	Quantity (ml)	Clinical impact
An approximation not necessarily referring to blood only, but to fluid collected from the abdomen during operation (blood, bile, peritoneal fluid/ascites) when a clear distinction cannot be made	(Suction fluids – irrigation fluids) + (operative gauze weight – dry gauze weight) (1 g = 1 ml)	Null	0–100	No intraoperative transfusion
		A	101–500	No intraoperative transfusion
		B	> 500	With or without intraoperative transfusion
		C	Any	Intraoperative adverse event with life-threatening complication, transfusion needed

## Discussion

The present E-AHPBA consensus provides a definition, standard estimation method, and grading for intraoperative blood loss in HPB surgery. With implementation of standardization in all fields of HPB surgery, from surgical procedures to learning curves, textbook outcomes, and surgical complications, the urgent need identified by the consensus was to standardize blood loss as an outcome metric. Historically, standardization of blood loss estimation encountered many obstacles, in general as in HPB surgery, owing to intrinsic technical limitations, cultural reluctance, and difficulty in accurately measuring blood loss as a whole^10,11^. Measured blood loss often represents an approximation, especially in open abdominal surgery, where hidden or difficult-to-measure losses and the loss of other types of fluids, such as ascites or bile, are involved, and all direct measurements lead to some degree of overestimation or underestimation of blood loss^15–17^. Although these difficulties remain, corrective factors can be applied whenever possible, such as subtracting −25 ml/h after common bile duct division, or using different suction canisters for different fluids, if they are reported in the context of a standardized blood loss estimation. Among all available methods, a direct one prevailed as the new reference. Taken the lack of studies comparing existing methods, this was not necessarily deemed as superior, but represented the most accurate, easily replicated, time- and cost-effective among those commonly used^9^. Indirect solutions, such as blood count-related formulas, are useful for research purposes and to complement direct estimation, which they shall not replace^15,18^. Similarly, existing bleeding scales designed to measure bleeding from a specific site at a specific time point (VIBe scale recently validated for liver surgery^14^) can be used to assess the effectiveness of haemostatic agents or surgical devices in clinical trials, without replacing or overlapping with general estimation of whole blood loss.

The most critical aspect of blood loss is represented by its potential association with perioperative outcomes and survival. It has been demonstrated, however, that most of the existing literature exploring this relationship is biased by inconsistent and inaccurate blood loss estimation^9^. For this reason, the present consensus expressed only general concern regarding the association between blood loss and postoperative complications after pancreatic and major and minor liver surgery, leaving it to future studies designed in compliance with a standardized method to further explore the relationship of blood loss with specific complications and long-term outcomes. Finally, the consensus proposed blood loss grading, mixing quantitative categories with qualitative factors (for example, requiring transfusions, or clinically threatening to the patient). Despite being subordinate to the treating clinician’s judgement^19^, this represents an attempt to increase accuracy with regard to clinical impact when dealing with severe bleeding, rather than pursuing cut-off measures exclusively. Of note, the presence of qualitative factors upscaling a patient to a higher blood loss grade does not cancel the need for quantitative reporting, allowing for future studies comparing outcomes and accounting for qualitative, quantitative, and patient factors (for example, a B-grade patient with blood loss 400 ml and coronary artery disease requiring transfusion may differ from a patient with blood loss 1000 ml not requiring transfusion).

All the statements included in this consensus reached the agreement threshold after the first round. Besides a high level of agreement between experts, this may also reflect a high degree of homogeneity among them. Indeed, an important limitation of the present consensus is that the project uniquely included surgeons who were members of the same society. Despite the exquisite surgical pertinence of many of the included statements, blood loss reporting and estimation is also common practice among anaesthetists. Surgeons and anaesthetists should share decision-making in response to significant bleeding.

## Collaborators

Members of the E-AHPBA Consensus Group on Blood Loss in Hepatopancreatobiliary Surgery: Mohamad Abu Hilal; Mustapha Adam; Luca Aldrighetti; Bodil Andersson; Angelakoudis Apostolos; Somaiah Aroori; Anita Balakrishnan; Domenico Bassi; Andrea Belli; Giammauro Berardi; Frederik Berrevoet; Marc Besselink; Maximilian Bockhorn; Ugo Boggi; René Borscheid; Stefan A. W. Bouwense; Raffaele Brustia; Carljin Buis; Sean Burmeister; Olivier Busch; Umberto Cillo; Ahmet Coker; Kevin Conlon; Stefano Crippa; Francesco E. D'Amico; Bobby V. M. Dasari; Raffaele De Luca; Roeland F. De Wilde; Christos Dervenis; John Devar; Marcello Di Martino; Safi Dokmak; Ela Ekmekcigil; Jennie Engstrand; Alessandro Esposito; Asmund Fretland; Isabella Frigerio; Tom Gallagher; Georgios Gemenetzis; Stefan Gilg; Francesco Giovinazzo; Brian K. P. Goh; Martina Guerra; Rachel Guest; Aiste Gulla; Andrew A. Gumbs; Thilo Hackert; Julie Hallet; Fiona Hand; Daniel Hartmann; Andrew J. Healey; Stefan Heinrich; Emir Hoti; Povilas Ignatavicius; Nigel Jamieson; Laukkarinen Johanna; Ed Jonas; Elio Jovine; Ben Jugmohan; Ambareen Kausar; Elie Keli; Zafar Khan; Jakob Kirkegard; Jorg Kleeff; Philipp Kron; Francesco Lancellotti; Sven Lang; Uttam Laudari; Johanna Laukkarinen; Michael Linecker; Victor Lopez; Hassan Z. Malik; Alessio Marchetti; Guillaume Martel; Emmanuel Melloul; Chistoph Michalski; Sanjay Pandanaboyana; Ioannis Passas; Julie Perinel; Dejan Radenkovic; Jose M. Ramia; Elena Rangelova; Niki Rashidian; Francesca Ratti; Artur Rebelo; Rami Rhaiem; Fernando Rotellar; Eran Sadot; Ville Sallinen; Tsaramanidis Savvas; Moritz Schmelzle; Alejandro Serrablo; Mario M. Serradilla; Olivia Sgarbura; Erik Shadde; Kjetil Soreide; Carlo Sposito; Stefan Stattner; Gregor A. Stavrou; Hanna Sternby; Oliver Strobel; Christian Sturesson; Lulu Tanno; Michele Tedeschi; Guido Torzilli; Gregory Tsiotos; Georgios Tsoulfas; Patricia S. Velazquez.

## Data Availability

Study data are available on request from the authors.
